# Supplementary material for: Searching for best lower dimensional visualization angles for high dimensional RNA-Seq data
Source: PeerJ. 2018 Jul 12;6:e5199. doi: 10.7717/peerj.5199 (PMC6046202; doi:10.7717/peerj.5199)
Supplement: Supplemental Information 1 [file peerj-06-5199-s001.pdf]

A. CONSTRUCTION OF COVARIANCE ELLIPSES FOR NORMAL COMPONENTS

In this section, we introduce how the covariance ellipses are constructed by MclustDA when a scatterplot or a scatterplot matrix is graphed.

For 2D data, suppose the mean and covariance estimates for component k of class j are $\hat{\mu}_{jk}$ and $\hat{\Sigma}_{jk}$, respectively. Also suppose that $\hat{\Sigma}_{jk}$ has eigenvalues $\lambda_1 \geqslant \lambda_2$ and their corresponding eigenvectors $\boldsymbol{e}_1$ and $\boldsymbol{e}_2$. Then MclustDA computes the major and minor axes of the ellipse centered at $\hat{\mu}_{jk}$ the following way:

$$\text{major axis} = \hat{\mu}_{jk} \pm \sqrt{\lambda_1}\boldsymbol{e}_1, \quad \text{minor axis} = \hat{\mu}_{jk} \pm \sqrt{\lambda_2}\boldsymbol{e}_2,$$

and the resulting ellipse has coverage probability of approximately 0.393.

In the case of higher dimensional data, MclustDA constructs the scatterplot and graphs the ellipses two dimensions at a time. Suppose $\hat{\mu}_{jk}$ and $\hat{\Sigma}_{jk}$ are defined the same way as above, and consider data dimensions p and q for visualization via scatterplot. Let $\Sigma^{(p,q)} = [\hat{\Sigma}_{jk}]_{(p,q)}$ be the covariance submatrix corresponding to the two dimensions, and $\mu^{(p,q)} = [\hat{\mu}_{jk}]_{(p,q)}$ be the corresponding mean vector. Now, suppose $\Sigma^{(p,q)}$ has eigenvalue/eigenvector pairs $\{\lambda_1^{(p,q)}, \boldsymbol{e}_1^{(p,q)}\}$ and $\{\lambda_2^{(p,q)}, \boldsymbol{e}_2^{(p,q)}\}$ with $\lambda_1^{(p,q)} \geqslant \lambda_2^{(p,q)}$. Then the ellipse plotted by MclustDA has major and minor axes as follows:

$$\text{major axis} = \mu^{(p,q)} \pm \sqrt{\lambda_1^{(p,q)}}\boldsymbol{e}_1^{(p,q)}, \quad \text{minor axis} = \mu^{(p,q)} \pm \sqrt{\lambda_2^{(p,q)}}\boldsymbol{e}_2^{(p,q)},$$

where the ellipse has the same coverage probability as the case above.

B. SCATTERPLOTS AND SCATTERPLOT MATRICES FOR SELECT TOP RANKED FEATURE COMBINATIONS

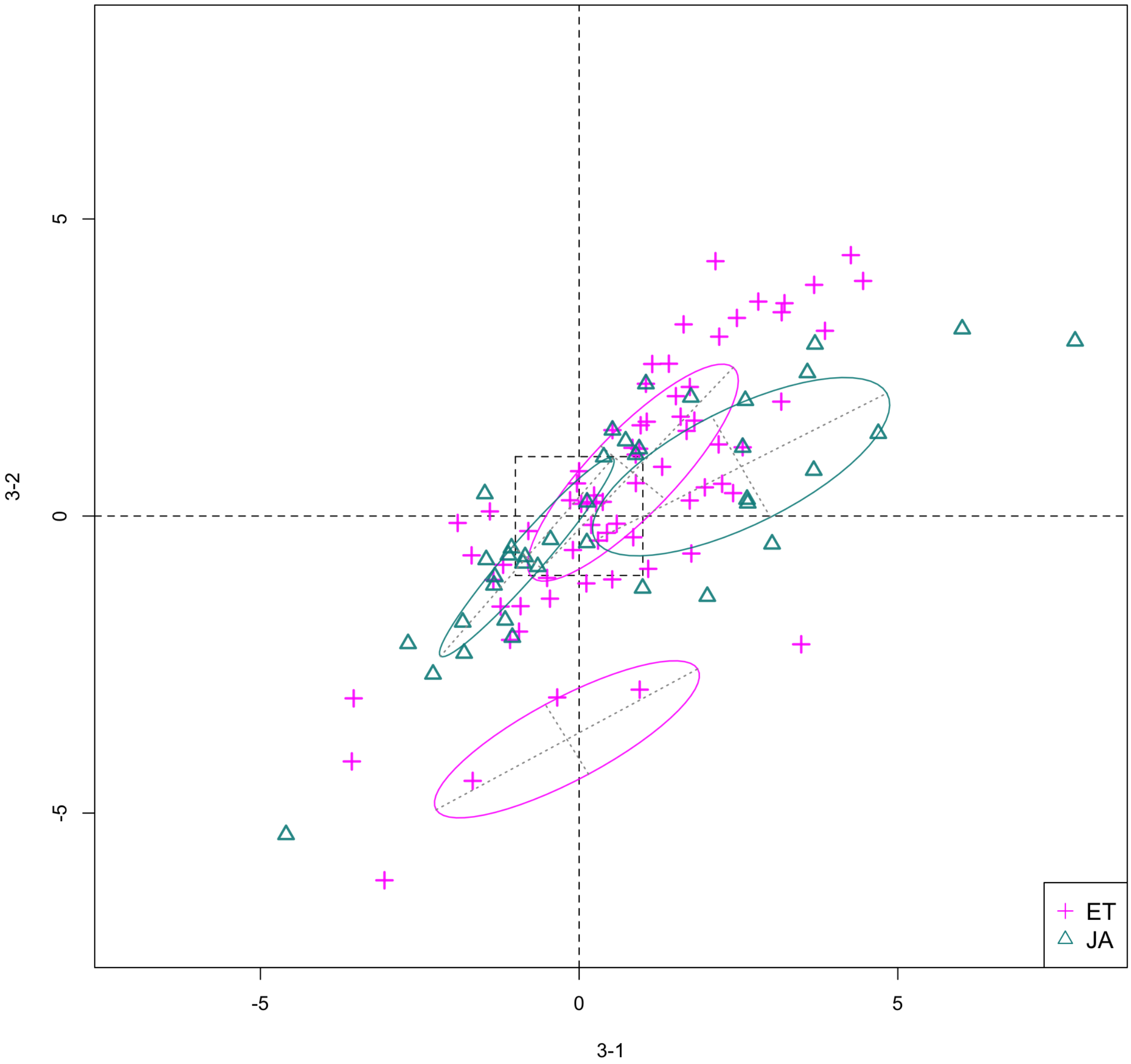

Figure A1: Scatterplot for 3-1 and 3-2

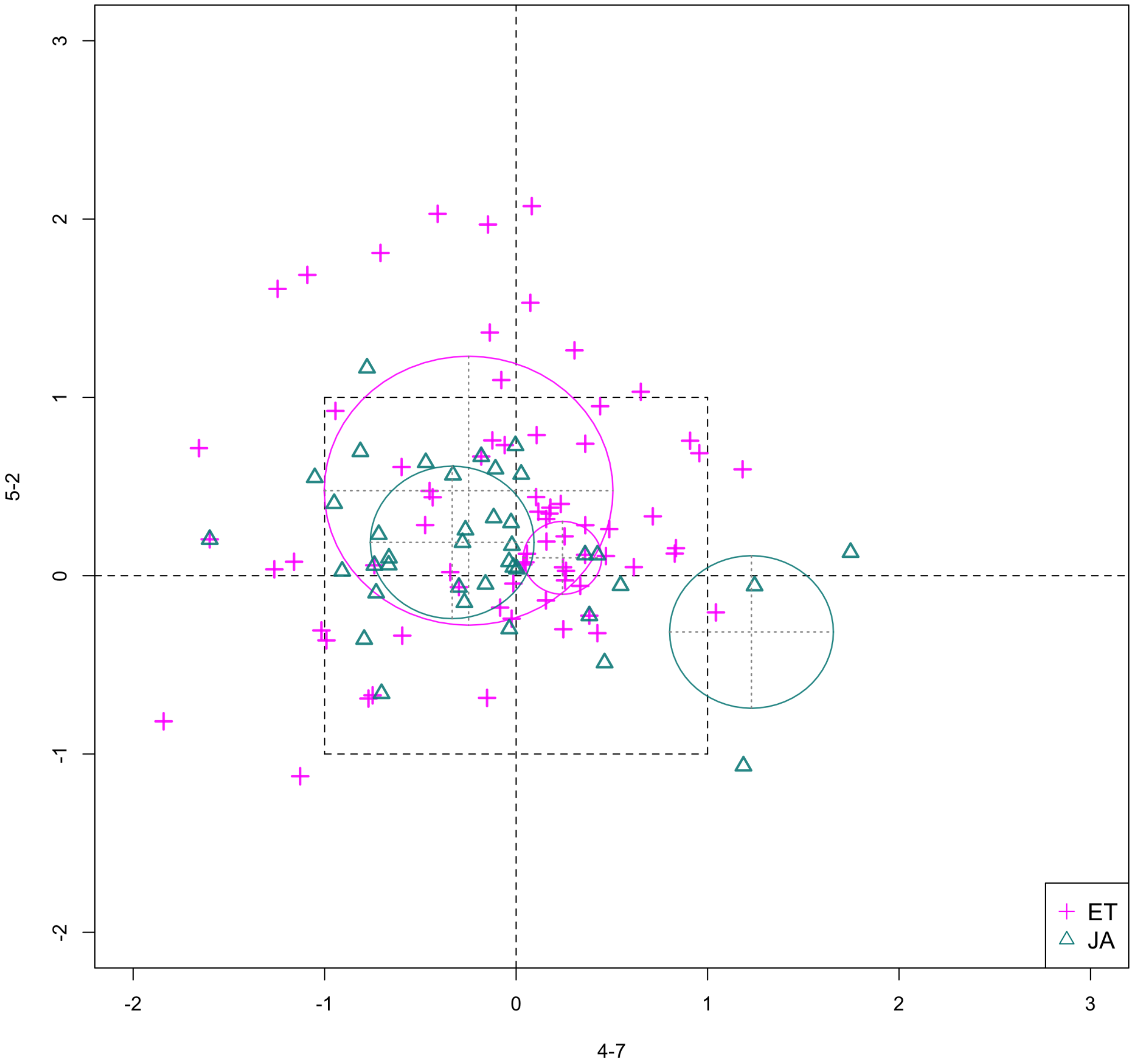

Figure A2: Scatterplot for 4-7 and 5-2

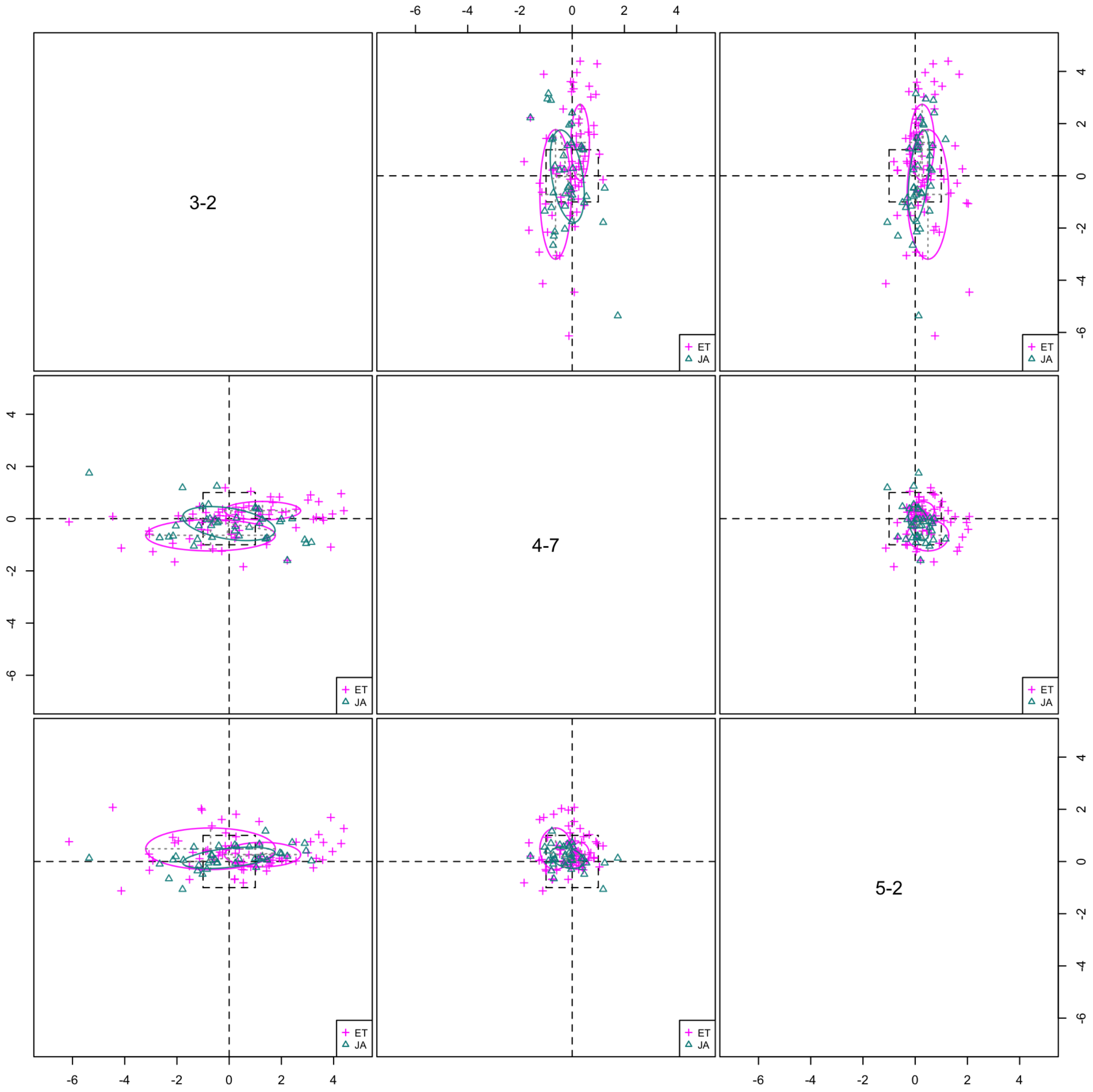

Figure A3: Scatterplot matrix for [3-2, 4-7, 5-2]

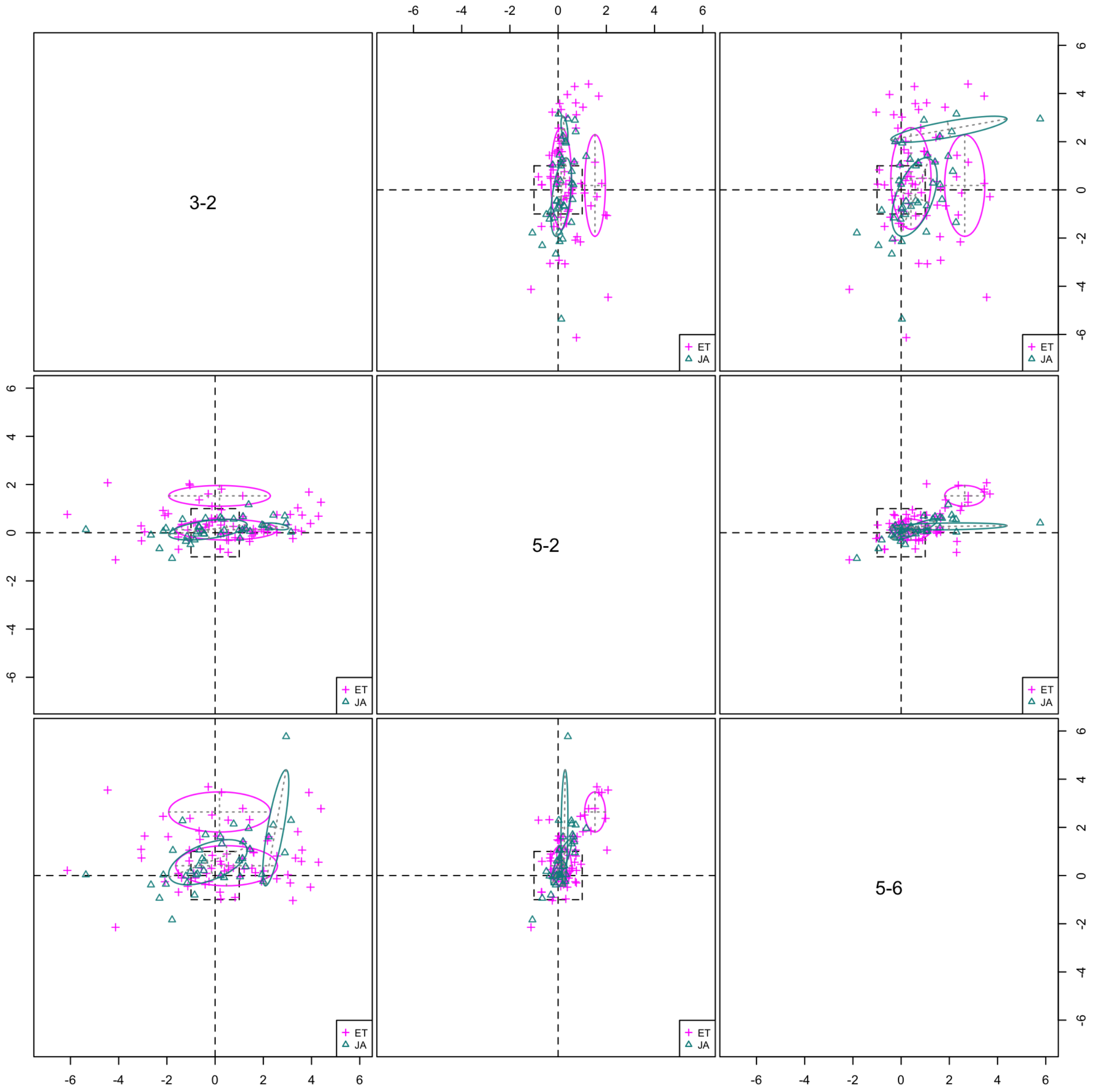

Figure A4: Scatterplot matrix for [3-2, 5-2, 5-6]

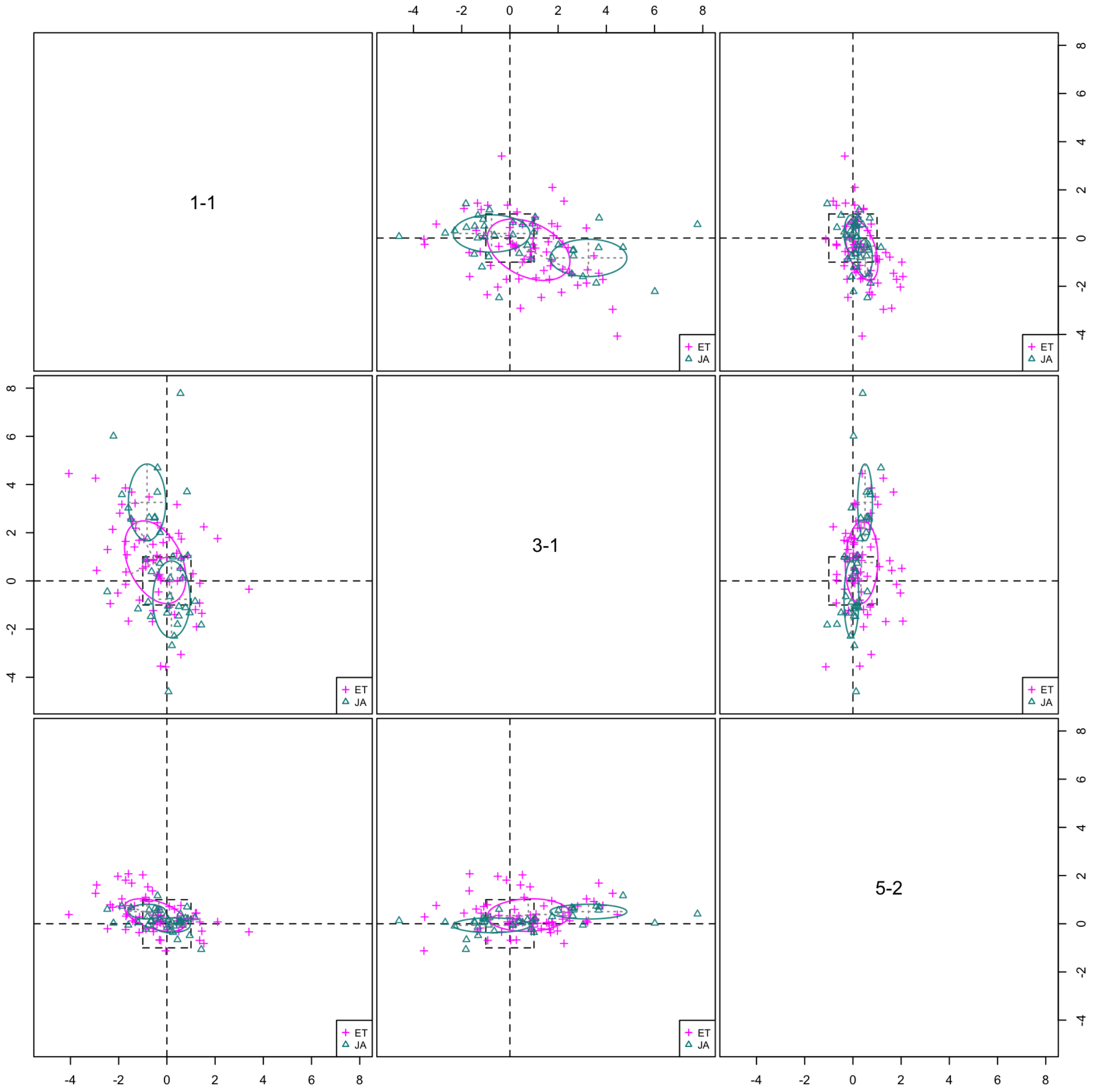

Figure A5: Scatterplot matrix for [1-1, 3-1, 5-2]

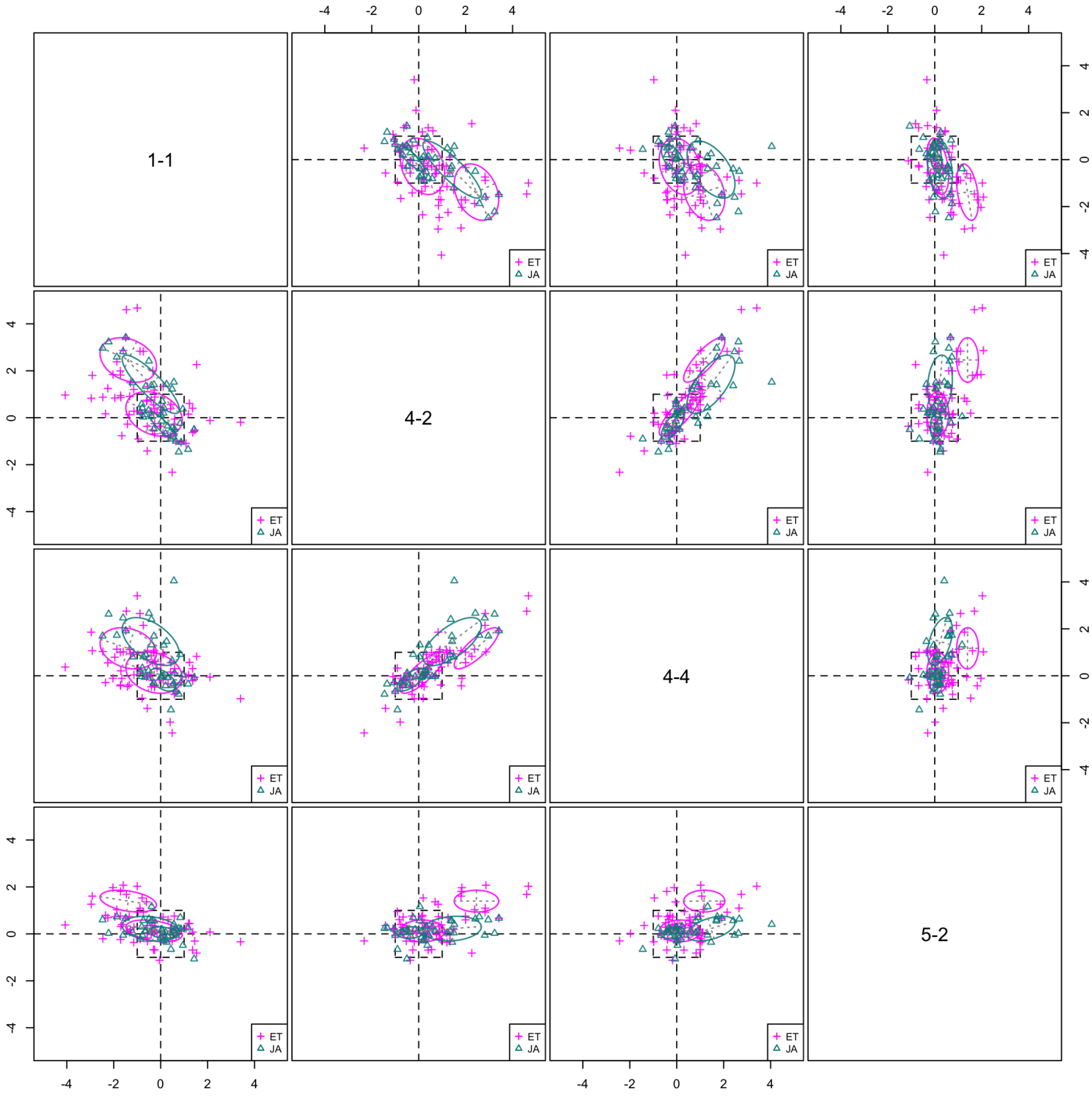

Figure A6: Scatterplot matrix for [1-1, 4-2, 4-4, 5-2]

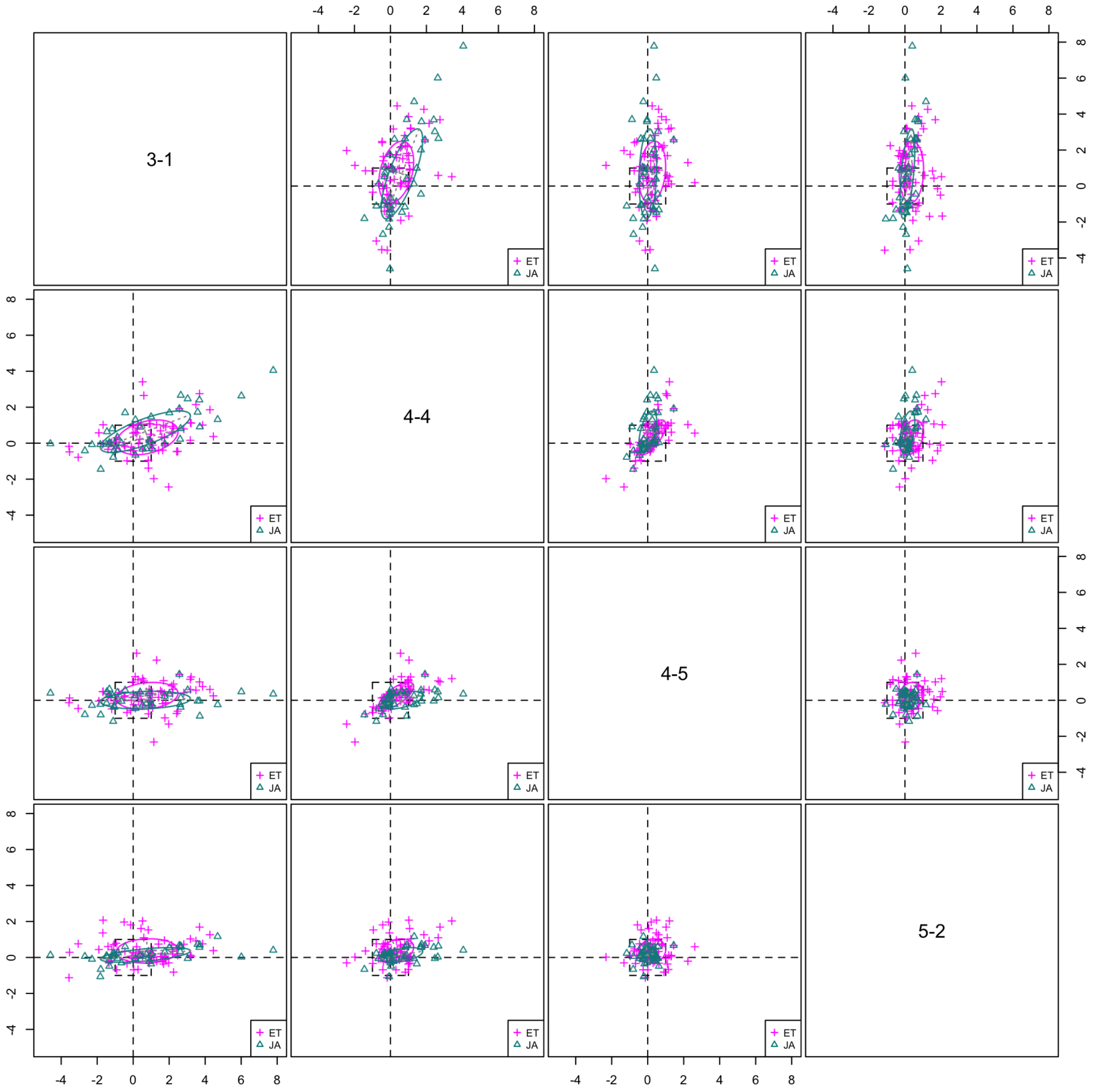

Figure A7: Scatterplot matrix for [3-1, 4-4, 4-5, 5-2]

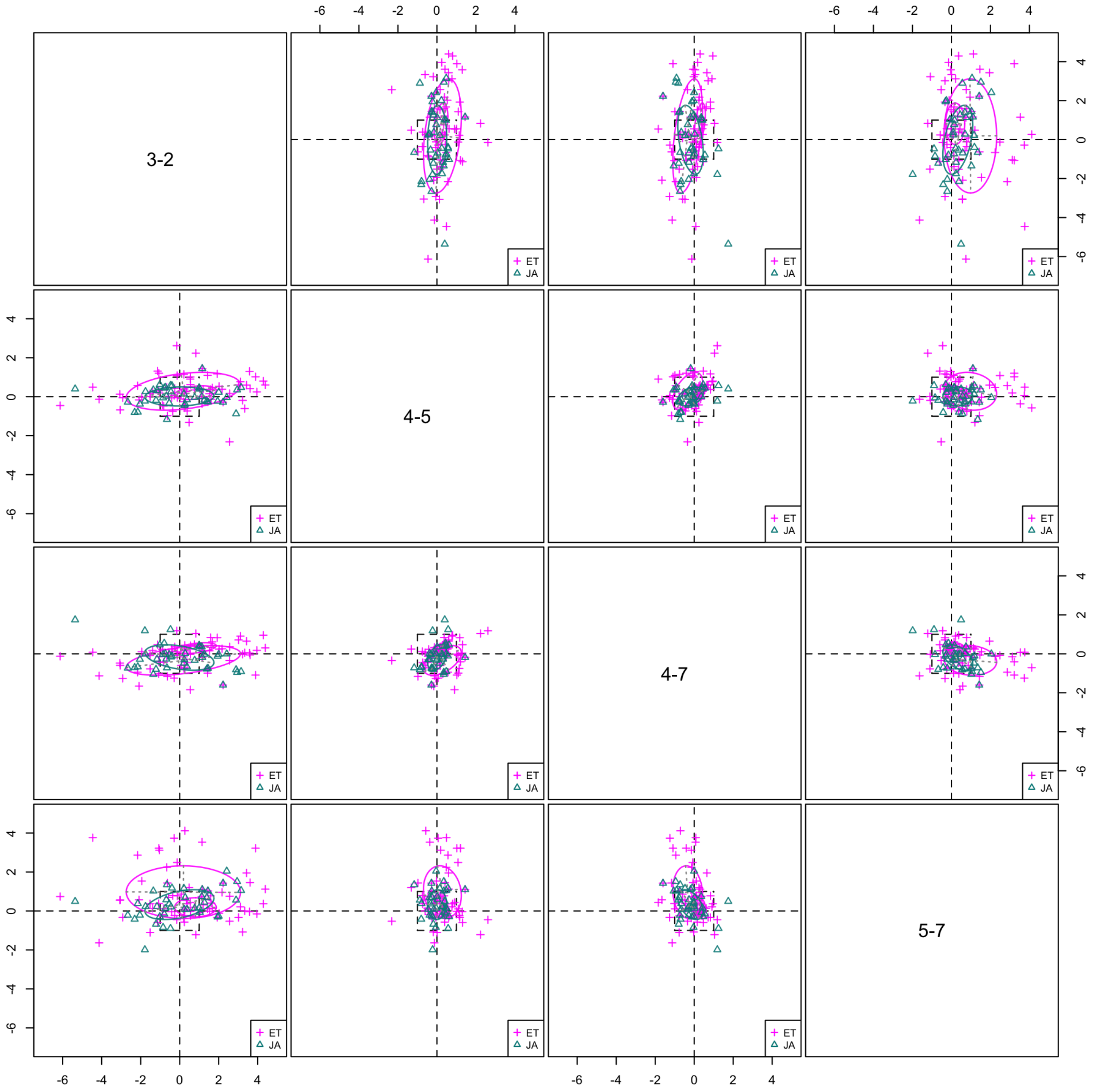

Figure A8: Scatterplot matrix for [3-2, 4-5, 4-7, 5-7]

C. BIASEDNESS AND VARIABILITY OF GSI PRODUCED BY REPEATED CV FOR LARGE SAMPLES

The main purpose of CV is to prevent overfitting and to correct the biased estimation of misclassification error rate, while repetition of CV is performed to avoid any potentially "bad" partitioning of the data in CV process.

To test the necessity of CV and repeated CV for large sample size, we performed

simulation studies to compare GSI values produced with and without CV/repeated CV for large, simulated data. Steps of the simulation are as follows:

1. Choose a feature pair $[i,\, j]$, and subset the data accordingly.

2. Fit MclustDA model to obtain parameter estimates for each class $\hat{\Theta}_1$ and $\hat{\Theta}_2$. Denote sample size in each class as N_1 and N_2.

3. For each class, simulate multivariate normal or mixture of normal data using $\hat{\Theta}_1$ and $\hat{\Theta}_2$ as model parameters.

   – Simulate with sample sizes mN_1 and mN_2, with $m = 1, 2, 5, 8, 10$

4. For each simulated dataset and the original dataset, calculate GSI, first using 10×10 repeated CV and recording all 10 CV outputs, and then without using any CV.

5. Repeat steps $1 \sim 4$ for all feature pairs.

C.1 Test variability of cross-validation results for large sample size

As observed in Figure A9, as sample size of the simulated data increases, CV results become more stable between repetitions.

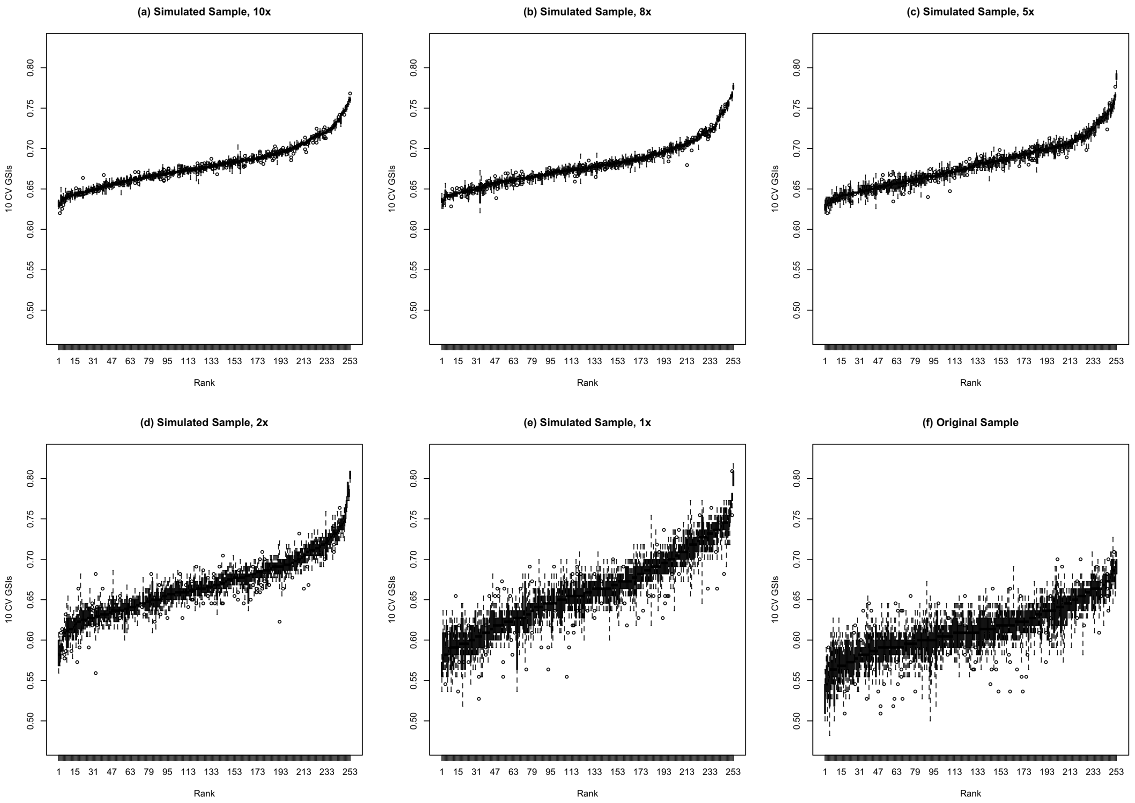

Figure A9: Variability of GSI values produced by repeated CV for simulated and original data. Feature pairs ranked by median GSI. All plots on the same scale.

C.2 Test necessity of CV for large dataset in terms of bias

As observed in Figure A10, for relatively small samples, GSI calculated without any cross-validation is often over-optimistic. The biasedness becomes ameliorated as sample size increases.

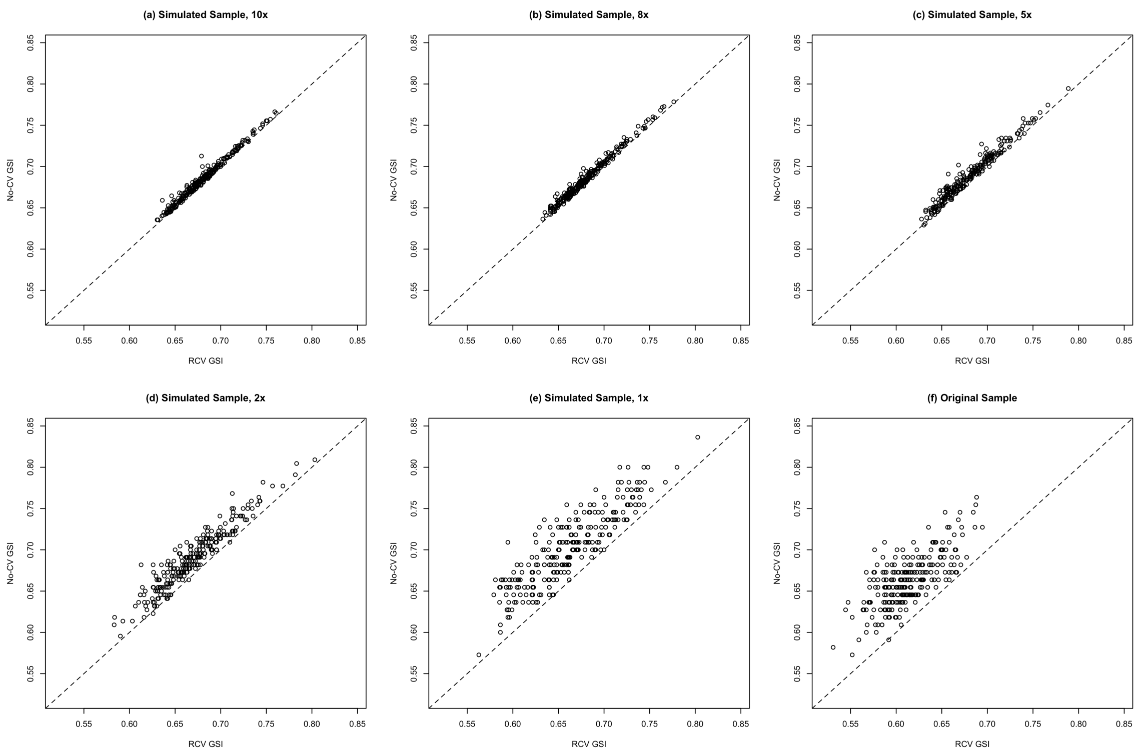

Figure A10: Comparision of GSI values produced with and without using RCV. All plots on the same scale.
